# Timing of acute kidney injury in infarction-related cardiogenic shock: early onset signals a high-risk phenotype – a retrospective observational study

**DOI:** 10.1186/s12882-025-04730-y

**Published:** 2026-01-06

**Authors:** Priyanka Boettger, Henriette Preusse-Sondermann, Jamschid Sedighi, Jannik Jobst, Hassan Hassan, Utku Bayram, Kerstin Piayda, Matthias Janusch, Birgit Assmus, Bernhard Unsoeld, Henning Lemm, Samuel Sossalla, Michael Buerke

**Affiliations:** 1 Department of Internal Medicine I, Cardiology, Critical Care Medicine, Justus-Liebig University, Giessen, Germany; 2 Department of Internal Medicine II, Cardiology, Angiology and Critical Care Medicine, St. Marien Hospital Siegen, Siegen, Germany; 3 Department of Internal Medicine II, Nephrology, Justus-Liebig University, Giessen, Germany; 4 Department of Cardiology, Kerckhoff Clinic, Campus Kerckhoff of the Justus-Liebig-University, Bad Nauheim, Germany; 5 Department of Health Policy, London School of Economics and Political Sciences, Houghton Street, WC2A2AE, London, United Kingdom

**Keywords:** Acute kidney injury, Cardiogenic shock, Myocardial infarction, AKI timing, Early-onset AKI, Hemodynamic instability, Lactate, Renal dysfunction, In-hospital mortality

## Abstract

**Background:**

Acute kidney injury (AKI) is common in cardiogenic shock (CS) and increases mortality, but the prognostic impact of onset timing in infarct-related CS is unclear. We examined whether early versus late AKI onset is associated with differences in patient characteristics and outcomes.

**Methods:**

In this retrospective observational study, 369 patients with infarct-related CS were classified by AKI timing within the first 96 h of admission: early (≤ 48 h) or late (> 48 h), according to KDIGO criteria. Clinical, hemodynamic, and inflammatory parameters and outcomes were compared. Multivariable logistic regression identified independent predictors of early AKI and in-hospital mortality.

**Results:**

AKI occurred in 143 patients (38.8%), with 56.6% early-onset. In-hospital mortality was higher with early AKI than late AKI (71.6% vs. 54.8%; absolute difference 16.8%, 95% CI 3.1–30.5; *p* = 0.018). Early AKI patients had higher lactate at admission (median 4.3 vs. 3.1 mmol/L; *p* = 0.028), greater norepinephrine requirements (0.34 vs. 0.21 µg/kg/min; *p* = 0.044), and more frequent mechanical ventilation (81.5% vs. 61.3%; *p* = 0.011). In multivariable analysis, early AKI independently predicted in-hospital mortality (adjusted OR 2.12, 95% CI 1.16–3.87; *p* = 0.015), and was associated with baseline creatinine (OR 5.68 per 1 mg/dL, *p* = 0.008) and 24-h lactate (OR 2.67 per mmol/L, *p* < 0.001).

**Conclusions:**

In infarct-related CS, AKI within 48 h marks a high-risk hemodynamic phenotype with markedly increased mortality, driven by renal vulnerability and early hypoperfusion. Incorporating AKI timing into risk stratification may help target early renoprotective interventions. Keywords: Acute kidney injury; cardiogenic shock; myocardial infarction; AKI timing; early-onset AKI; hemodynamic instability; lactate; renal dysfunction; in-hospital mortality

## Introduction

Cardiogenic shock (CS) secondary to acute myocardial infarction remains a clinical emergency with persistently high mortality, despite advances in early revascularization, mechanical circulatory support, and critical care protocols [1, 2]. Defined by inadequate tissue perfusion due to reduced cardiac output, CS initiates a cascade of vasoconstriction, neurohormonal activation, systemic inflammation, and ultimately multi-organ failure [1].

Among extracardiac complications, the kidney is particularly vulnerable. Acute kidney injury (AKI) occurs frequently in CS and is associated with longer Intensive care unit (ICU) stay, increased need for renal replacement therapy (RRT), and excess mortality [3]. The pathophysiology is multifactorial, involving renal hypoperfusion, venous congestion, systemic inflammation, nephrotoxins, and metabolic stress [4, 5]. Recent studies have highlighted the clinical and biological heterogeneity of AKI in critical illness, particularly with respect to timing of onset. Early AKI—typically defined as occurring within the first 48 h—often reflects primary hemodynamic insult, while late AKI, developing beyond 48 h, is more frequently associated with secondary injury mechanisms such as sepsis, cumulative vasopressor exposure, nephrotoxins, and multiorgan dysfunction [6, 7]. Beyond these temporal distinctions, recent work has advanced AKI phenotyping by integrating clinical and molecular features. Plasma and urinary biomarkers such as Neutrophil gelatinase–associated lipocalin (NGAL), kidney injury molecule-1 (KIM-1), interleukin-18 (IL-18), cystatin C (CysC), and the combined cell-cycle arrest biomarker tissue inhibitor of metalloproteinases-2 · insulin-like growth factor–binding protein 7 (TIMP-2·IGFBP7) allow early detection of renal stress and stratify patients according to inflammatory and epithelial injury pathways [8–12]. Large multicenter registries, including FINNAKI [13] and AKI-EPI [14], have shown that these biological sub phenotypes are consistently linked to mortality, chronic kidney disease progression, and cardiovascular events [15–17]. The Kidney Health Initiative has therefore proposed incorporating biomarker-guided phenotyping into future AKI definitions to improve risk prediction [8, 18–20]. While biomarker-based approaches refine biological AKI subtypes, the temporal dimension remains clinically pivotal, as timing of onset may reflect distinct underlying mechanisms and hemodynamic profiles.

Patients with early AKI often have pre-existing renal impairment and elevated lactate levels, indicating systemic hypoperfusion [21], whereas late AKI is more common in older, multimorbid patients and is frequently irreversible [22].

Importantly, both early and late AKI are associated with increased mortality, but late AKI confers particularly poor outcomes, including higher risk of death, arrhythmias, bleeding, and longer ICU stay [23–25]. Late AKI also contributes to persistent cardiovascular dysfunction through maladaptive remodeling and sympathetic overstimulation, and predisposes survivors to chronic kidney disease and recurrent heart failure [21, 26]. Despite these insights, the prognostic relevance of AKI timing has not been systematically examined in infarct-related CS [27]. Existing AKI classifications, including the Kidney Disease: Improving Global Outcomes (KDIGO) do not distinguish between early and late phenotypes, and few studies have explored whether early-onset AKI predicts mortality independently of renal function or shock severity at admission. The aim of this study was therefore to investigate the timing of AKI onset in infarct-related CS and to examine whether the time of development is associated with differences in patient characteristics, treatment intensity, and clinical outcomes, including in-hospital mortality. To address this gap, we focused exclusively on patients with infarct-related CS, thereby providing a homogeneous population to evaluate the prognostic implications of AKI timing within the acute shock phase.

## Methods

**Study design and population** We conducted a retrospective, single-center observational study at an academic hospital in Germany. Consecutive patients admitted to the intensive care unit with cardiogenic shock between January 2010 and 2015 were screened for eligibility. This cohort has been previously described in analyses addressing different research questions; the present study focuses specifically on the prognostic relevance of AKI timing in infarct-related cardiogenic shock [28].

**Study objectives** The *primary objective* was to determine whether early-onset AKI (≤ 48 h) was associated with a higher in-hospital mortality compared with late-onset AKI (> 48–96 h) in infarct-related cardiogenic shock.

*Secondary objectives* were to compare early- versus late-onset AKI with respect to.

AKI severity (KDIGO stages 1–3), lactate levels, vasopressor requirements, need for renal replacement therapy, and mechanical ventilation.

### Exposure and outcome definitions

The exposure of interest was the timing of AKI onset, defined as:

- early AKI: ≤48 h.
- late AKI: >48–96 h.

Patients who did not develop AKI within the first 96 h served as the reference group for outcome comparisons.

### Inclusion criteria

Patients were included if they met all of the following:

1. **Infarct-related cardiogenic shock** [29], defined by.
  systolic blood pressure < 90 mmHg for ≥ 30 min or requirement for vasopressor support,
  signs of end-organ hypoperfusion (e.g., oliguria, altered mental status) [30], and.
  diagnosis of acute myocardial infarction according to contemporary ESC and AHA/ACC guidelines [31, 32].
2. Availability of serial serum creatinine measurements during the first 96 h of ICU admission, enabling accurate classification of AKI onset.

### Exclusion criteria

Patients were excluded if they met any of the following:

1. **Cardiogenic shock not attributable to acute myocardial infarction** (i.e., non–infarct-related cardiogenic shock, such as acute decompensated heart failure, myocarditis, acute valvular disease, or arrhythmia-related shock without myocardial infarction) (*n* = 14).
2. **Insufficient laboratory or clinical data** to permit KDIGO-based AKI classification or assignment of AKI onset timing within the prespecified 96-hour window (*n* = 8).

**Fig. 1:**
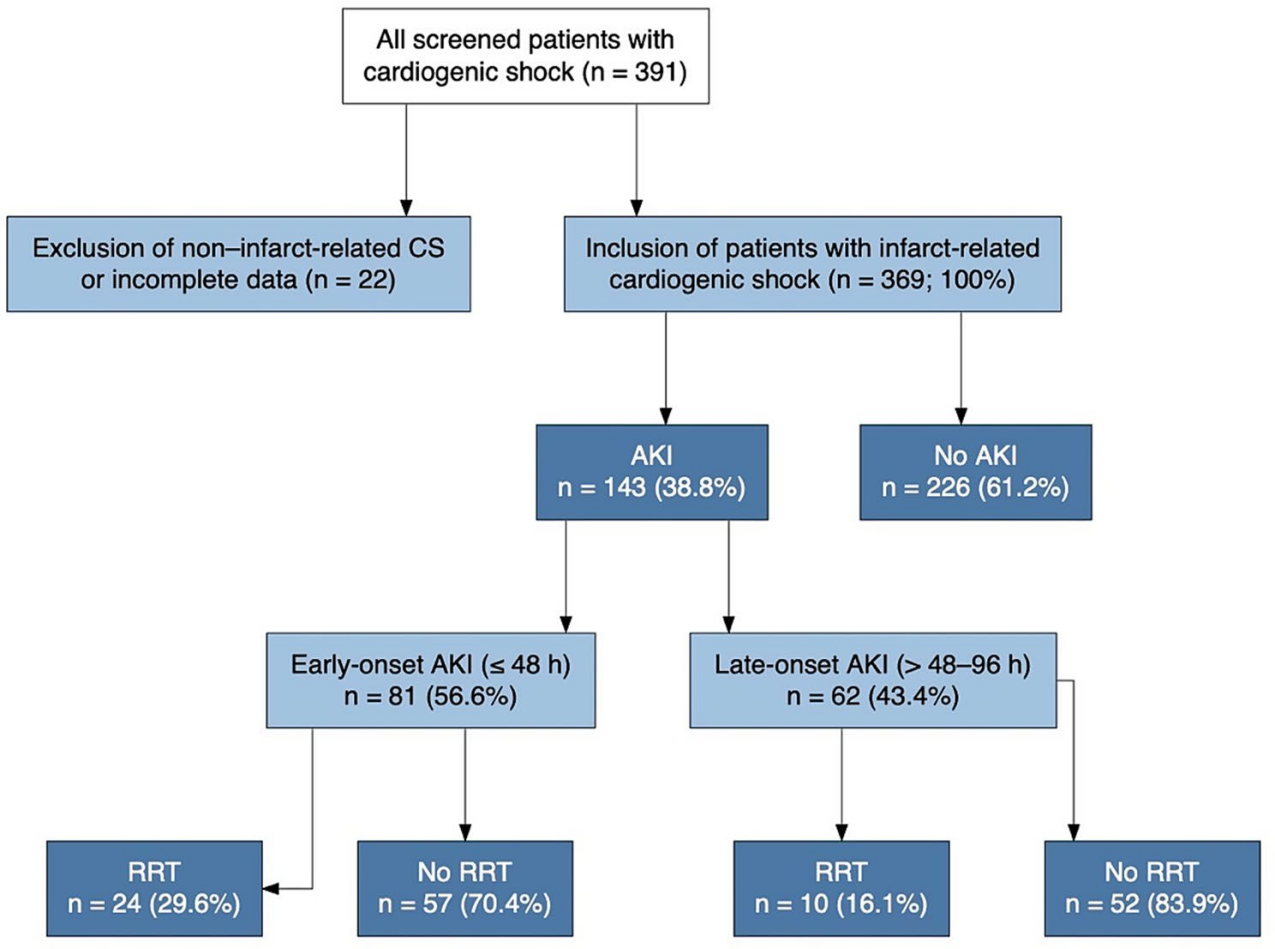
Study flow diagram and classification by AKI timing. Consecutive patients with cardiogenic shock were screened (*n* = 391). After exclusion of non–infarct-related cardiogenic shock and incomplete data (*n* = 22), 369 patients with infarct-related cardiogenic shock were included. Acute kidney injury (AKI) within the first 96 h occurred in 143 patients (38.8%), while 226 patients (61.2%) did not develop AKI. AKI was classified as early-onset (≤ 48 h; *n* = 81, 56.6%) or late-onset (> 48–96 h; *n* = 62, 43.4%). Use of renal replacement therapy (RRT) within each AKI timing group is shown

**Data collection and variables** All patients with cardiogenic shock (CS) admitted between January 2010 and July 2015 to an academic tertiary-care hospital in Germany were screened. A total of 391 patients were evaluated, of whom 22 were excluded because of non–infarct-related CS (*n* = 14) or incomplete data (*n* = 8). The final cohort comprised 369 patients with infarct-related CS. Among these, 143 patients (38.8%) developed acute kidney injury (AKI) within the first 96 h of ICU admission, consistent with the proportions shown in Fig. 1. All patients underwent coronary angiography and, if indicated, PCI using low-osmolality iodinated contrast. Periprocedural hydration with isotonic saline was performed in accordance with institutional protocol. No contrast-enhanced CT imaging was performed during the initial ICU phase. Baseline demographics, comorbidities, laboratory parameters, hemodynamic data, and therapeutic interventions were recorded prospectively using an electronic case report form. Particular attention was paid to renal function dynamics, vasopressor use, and respiratory support. Serum creatinine was measured at least daily throughout the ICU stay. Baseline creatinine was defined as the most recent outpatient value within the preceding 12 months; if unavailable, the admission creatinine was used.

**AKI definition** AKI was defined and staged according to the Kidney Disease: Improving Global Outcomes (KDIGO) criteria [33], applying serum creatinine (SCr)–based thresholds. SCr was measured at least once daily in all patients and, in cases of oliguria, remeasured up to four times per day (14 patients; 3.8%) to ensure early detection and consistent classification. Urine output (UO) was recorded hourly and used for subanalyses but was not incorporated into the primary AKI definition because of its susceptibility to measurement variability and the confounding effects of diuretic therapy [9, 34]. In addition, complete KDIGO-conform urine output documentation over sustained 6-hour windows was not available for the entire retrospective cohort; therefore, UO criteria were not used for the primary timing classification of AKI. Baseline SCr was defined as the first ICU value, in accordance with KDIGO guidance when pre-admission data are unavailable [35, 36], a method validated in critically ill populations [37, 38]. To evaluate the prognostic impact of AKI timing, patients developing AKI within the first 96 h of ICU admission were categorized as early (≤ 48 h) or late (> 48–96 h) onset. The 96-hour window was chosen to represent the acute shock phase, during which renal dysfunction most likely reflects hemodynamic instability rather than subsequent ICU-related complications. Patients without AKI during this period served as the reference group.

**Renal replacement therapy** RRT was initiated in accordance with international guideline recommendations [39–41]based on the presence of refractory metabolic acidosis (pH < 7.1), severe hyperkalemia (K⁺ > 6.0 mmol/L) unresponsive to medical therapy, persistent anuria or oliguria (< 0.3 mL/kg/h for > 24 h), or volume overload leading to pulmonary congestion despite diuretic treatment. RRT was also initiated in cases of overt uremic complications such as encephalopathy or pericarditis. No patient received prophylactic or preemptive RRT in the absence of these clinical indications.

**Outcomes** The primary outcome was in-hospital mortality. Secondary outcomes included AKI severity (KDIGO stages 1–3), requirement for RRT, mechanical ventilation duration, and norepinephrine dosing over time.

### Statistical analysis

Analyses were performed using SPSS Statistics 24. Continuous variables are reported as means ± SD or medians (IQR), categorical variables as counts (%). Group differences were assessed using χ² or Fisher’s exact test for categorical variables and Student’s t-test or Mann–Whitney U test for continuous variables. Temporal trajectories of creatinine, lactate, and norepinephrine dose were examined by repeated-measures ANOVA. Survival was evaluated by Kaplan–Meier curves with log-rank testing, and Cox regression estimated the association between AKI timing and in-hospital mortality. A 1:1 propensity-score-matched comparison was performed to account for baseline imbalances. Predictors of early AKI and in-hospital mortality were analyzed using parsimonious multivariable logistic regression models. Covariates were selected a priori based on clinical relevance in cardiogenic shock and supplemented by variables with *p* < 0.10 in univariable testing. The final adjustment set comprised age, sex, diabetes, chronic kidney disease, baseline eGFR, previous myocardial infarction, left-ventricular ejection fraction, lactate on admission, use of mechanical circulatory support, vasopressor dose, and mechanical ventilation. To reduce small-sample bias and assess internal validity, Firth’s penalized regression and bootstrap resampling (1,000 iterations) were performed. Model discrimination was quantified using the area under the ROC curve. Missing data were handled by complete-case analysis. Two-sided *p* < 0.05 was considered statistically significant.

## Results

Of 391 patients screened for infarct-related cardiogenic shock, 369 met inclusion criteria and were analyzed (Fig. 1). Acute kidney injury (AKI) developed in 143 patients (38.8%); of these, 81 patients (56.6%) had early-onset AKI (≤ 48 h) and 62 (43.4%) late-onset AKI (> 48–96 h).

### Baseline characteristics by timing of AKI onset

Baseline demographics and major comorbidities were comparable across early and late AKI groups (all *p* > 0.05). Detailed characteristics are provided in Table 1. Markers of shock severity differed between groups: patients with early-onset AKI had higher baseline serum creatinine (*p* = 0.048), higher admission lactate (*p* = 0.032), and higher C-reactive protein (*p* = 0.038). Early AKI was also associated with more frequent mechanical ventilation (*p* = 0.011), indicating a more compromised physiological state at presentation.

**Table 1:** Baseline characteristics of the study population (*N* = 369)

Variable	Total cohort (*N* = 369)	Early AKI (*n* = 81)	Late AKI (*n* = 62)	*p*-value
Age, years (mean ± SD)	69.2 ± 12.2	69.9 ± 11.4	70.8 ± 9.2	0.56
Age group < 60 years	22.3% (82)	21.0% (17)	19.4% (12)	0.77
Age group 60–75 years	41.7% (153)	42.0% (34)	40.3% (25)	0.88
Age group > 75 years	36.0% (132)	37.0% (30)	40.3% (25)	0.71
Sex: Male	66.9% (247)	63.4% (51)	62.9% (39)	0.95
Sex: Female	33.1% (122)	36.6% (30)	37.1% (23)	0.95
Height, cm (mean ± SD)	171 ± 12.2	170 ± 11.8	171 ± 12.4	0.74
Weight, kg (mean ± SD)	80.6 ± 13.8	79.8 ± 13.1	81.0 ± 13.6	0.68
Body mass index, kg/m² (mean ± SD)	27.3 ± 4.4	27.4 ± 4.5	27.2 ± 4.3	0.81
Smoking history	45.7% (163)	44.4% (36)	46.8% (29)	0.79
Known coronary artery disease	31.4% (101)	24.7% (20)	22.6% (14)	0.77
Prior myocardial infarction	23.3% (74)	24.7% (20)	22.6% (14)	0.77
Previous CABG surgery	15.3% (49)	14.8% (12)	16.1% (10)	0.84
Chronic heart failure	33.6% (124)	34.6% (28)	30.6% (19)	0.63
Hypertension	75.4% (260)	76.5% (62)	75.8% (47)	0.91
Diabetes mellitus	52.4% (182)	50.6% (41)	54.8% (34)	0.63
Hyperlipoproteinemia	55.8% (193)	51.2% (41)	64.5% (40)	**0.034**
Obesity	63.8% (217)	67.9% (55)	66.1% (41)	0.82
Family history of CAD	33.7% (116)	32.1% (26)	33.9% (21)	0.81
Chronic kidney disease	84.8% (313)	87.7% (71)	82.3% (51)	0.45
KDIGO stage 3	35.6% (131)	37.0% (30)	34.0% (21)	0.72
KDIGO stage 5	1.8% (7)	2.5% (2)	1.6% (1)	0.62
Baseline creatinine (mg/dL, mean ± SD)	1.28 ± 0.40	1.34 ± 0.38	1.21 ± 0.41	**0.048**
Lactate at admission (mmol/L, median [IQR])	3.3 [2.3–5.0]	3.7 [2.8–5.3]	2.9 [2.1–4.6]	**0.032**
C-reactive protein (mg/dL, mean ± SD)	12.9 ± 6.9	14.2 ± 7.5	11.3 ± 6.2	**0.038**

Values are presented as mean ± standard deviation, median [interquartile range], or percentage (absolute number). Early AKI was defined as onset within ≤ 48 h of ICU admission; late AKI as onset > 48–96 h. P-values refer to comparisons between early and late AKI groups using chi-square or fisher’s exact test for categorical variables, and student’s t-test or Mann–Whitney U test for continuous variables, as appropriate. Bold p-values indicate statistical significance (*p* < 0.05)

### Hemodynamic and metabolic differences

Early-onset AKI was accompanied by more pronounced metabolic and circulatory compromise. Lactate concentrations were higher both at admission and at 24 h (both *p* = 0.032), and norepinephrine requirements at 24 h were significantly greater (*p* = 0.044). These findings are illustrated in Fig. 3 and emphasize the hemodynamic instability of early AKI.

### Temporal distribution of AKI onset

**Fig. 2:**
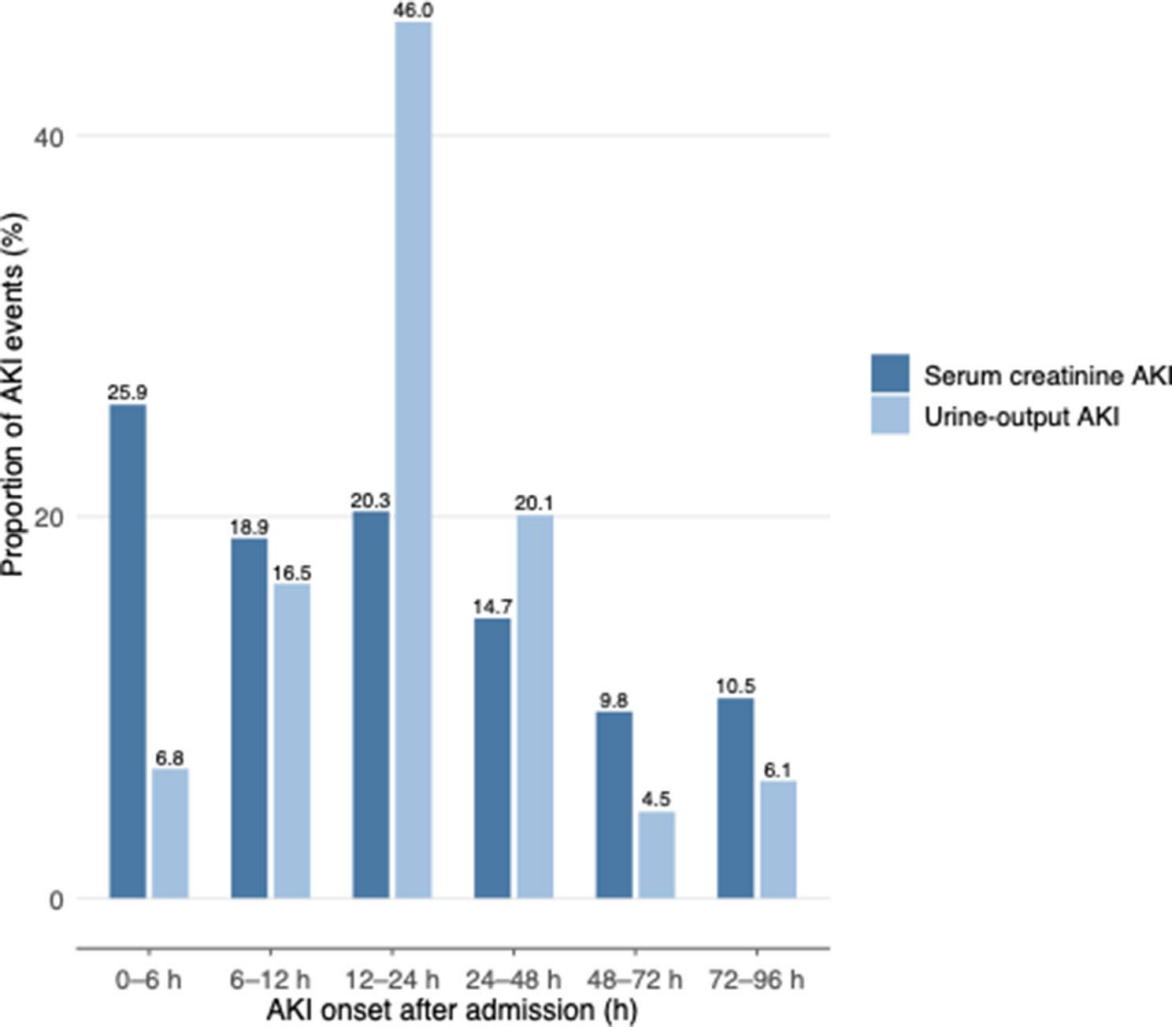
Distribution of AKI onset during the first 96 h after ICU admission in patients with infarct-related cardiogenic shock. AKI onset based on serum creatinine–defined KDIGO criteria is shown for the full AKI cohort (*n* = 143). Urine output–based AKI onset is displayed for a subset of patients with sufficient hourly urine output documentation to permit KDIGO window-based assessment and represents an exploratory analysis. Overall, AKI events clustered early during the acute shock phase, with the highest proportion occurring within the first 24 h after admission

**Fig. 3:**
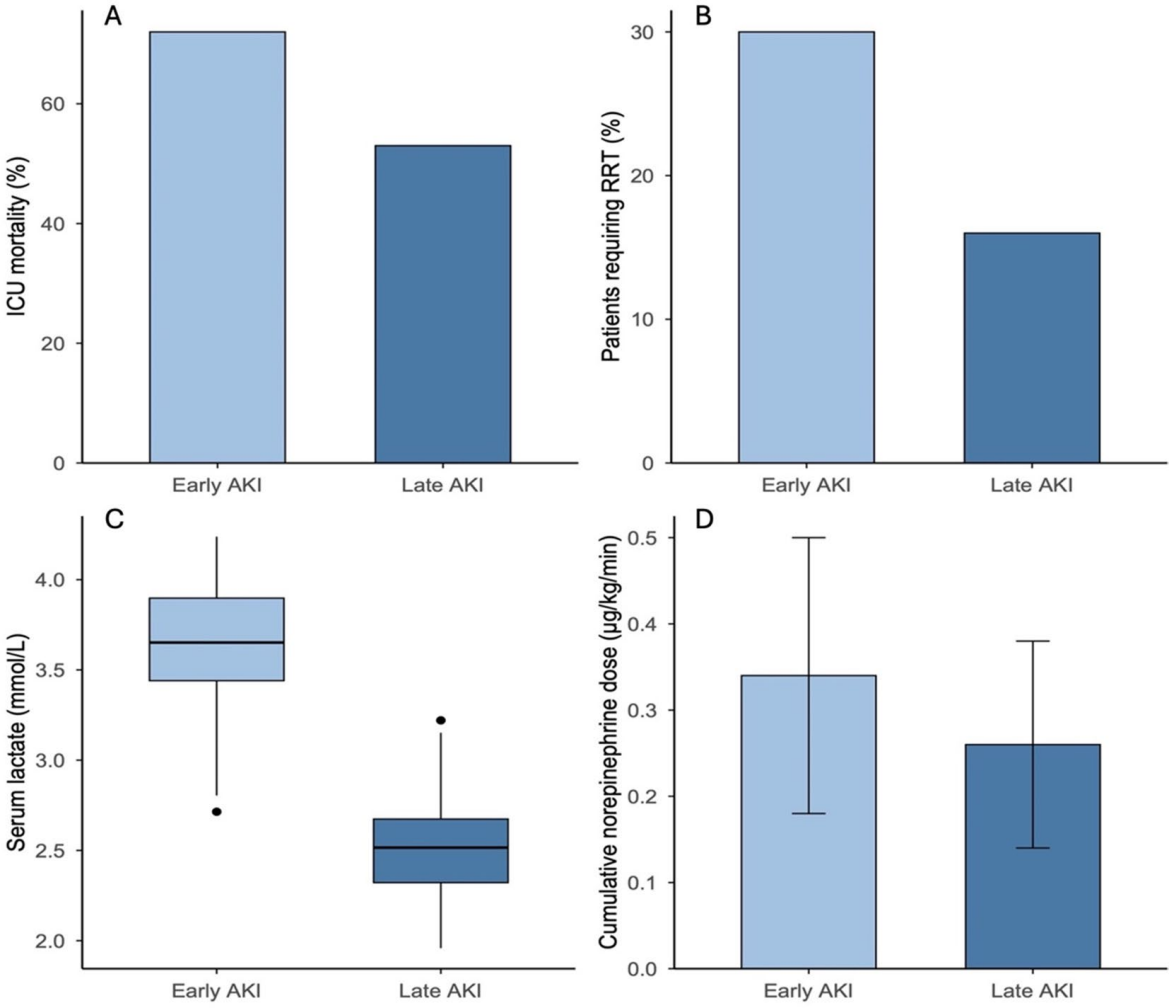
Outcomes stratified by timing of AKI in infarct-related cardiogenic shock. (**A**) ICU mortality was significantly higher in patients with early-onset AKI (≤ 48 h) compared with late-onset AKI (> 48–96 h) (71.6% vs. 54.8%; *p* = 0.018). (**B**) Requirement for RRT was more frequent in early- versus late-onset AKI (29.6% vs. 16.1%; *p* = 0.037). (**C**) Serum lactate concentrations at admission were elevated in early-onset AKI (median 3.7 [IQR 2.8–5.3] mmol/L) compared with late-onset AKI (2.9 [2.1–4.6] mmol/L; *p* = 0.032). (**D**) Mean norepinephrine dose at 24 h after ICU admission was higher in early-onset AKI (0.34 ± 0.14 µg/kg/min) than in late-onset AKI (0.27 ± 0.11 µg/kg/min; *p* = 0.044)

Most AKI episodes occurred early during the shock phase. Based on serum creatinine–defined KDIGO criteria, more than three quarters of all AKI events occurred within the first 24 h after ICU admission, with the highest frequency during the early shock phase. New AKI beyond 48 h was rare. This pronounced early clustering supports the predefined classification into early (≤ 48 h) and late (> 48–96 h) AKI (Fig. 2). Patients with early-onset AKI exhibited more severe initial shock. Baseline creatinine (*p* = 0.048), admission lactate (*p* = 0.032), and admission CRP (*p* = 0.038) were all higher in early compared with late AKI, and mechanical ventilation at presentation was more frequent (*p* = 0.011). Early AKI was further characterized by greater metabolic and circulatory compromise during the first 24 h, with higher lactate levels and higher norepinephrine requirements at 24 h (both *p* = 0.032 and *p* = 0.044, respectively; Fig. 3).

**Table 2:** Clinical outcomes stratified by timing of acute kidney injury (AKI)

Outcome	Total cohort (*N* = 369)	Early AKI (*n* = 81)	Late AKI (*n* = 62)	*p*-value
Mechanical ventilation	71.0% (262)	81.5% (66)	61.3% (38)	**0.011**
In-hospital mortality	57.7% (213)	71.6% (58)	54.8% (34)	**0.018**
30-day post-discharge mortality	1.1% (4)	1.2% (1)	0.9% (1)	0.81
Renal replacement therapy	13.6% (50)	29.6% (24)	16.1% (10)	**0.037**
Norepinephrine dose at 24 h (µg/kg/min)	0.31 ± 0.13	0.34 ± 0.14	0.27 ± 0.11	**0.044**
Lactate at admission (mmol/L)	3.3 [2.3–5.0]	3.7 [2.8–5.3]	2.9 [2.1–4.6]	**0.032**
Lactate at 24 h (mmol/L)	3.3 ± 1.7	3.7 ± 1.6	2.9 ± 1.3	**0.032**
Peak serum creatinine (mg/dL)	2.31 ± 1.2	2.41 ± 0.72	2.18 ± 0.65	**0.046**

Early AKI was defined as onset ≤ 48 h after ICU admission, and late AKI as onset > 48–96 h. Values are presented as percentages (n), means ± standard deviations, or medians with interquartile ranges, as appropriate. P-values refer to comparisons between the early- and late-onset AKI groups

### Clinical outcomes associated with AKI timing

Early-onset AKI was consistently associated with a more adverse in-hospital course. Mortality was significantly higher in early compared with late AKI (71.6% vs. 54.8%; *p* = 0.018), and early AKI patients required renal replacement therapy nearly twice as often (29.6% vs. 16.1%; *p* = 0.037). Across the entire cohort, the duration of invasive mechanical ventilation showed a markedly right-skewed distribution, with a median of 82 h (IQR 18–227 h). Patients with early-onset AKI required substantially longer ventilatory support than those with late-onset AKI (median 194 h [IQR 102–344] vs. 128 h [IQR 58–238]; *p* = 0.02).

Within the early AKI group, the subgroup requiring RRT had the poorest prognosis, with mortality exceeding 80% (83.3% vs. 64.7%; *p* = 0.041). These outcome differences are detailed in Table 2 and illustrated in Fig. 3.

### Predictors of early-onset AKI

**Fig. 4:**
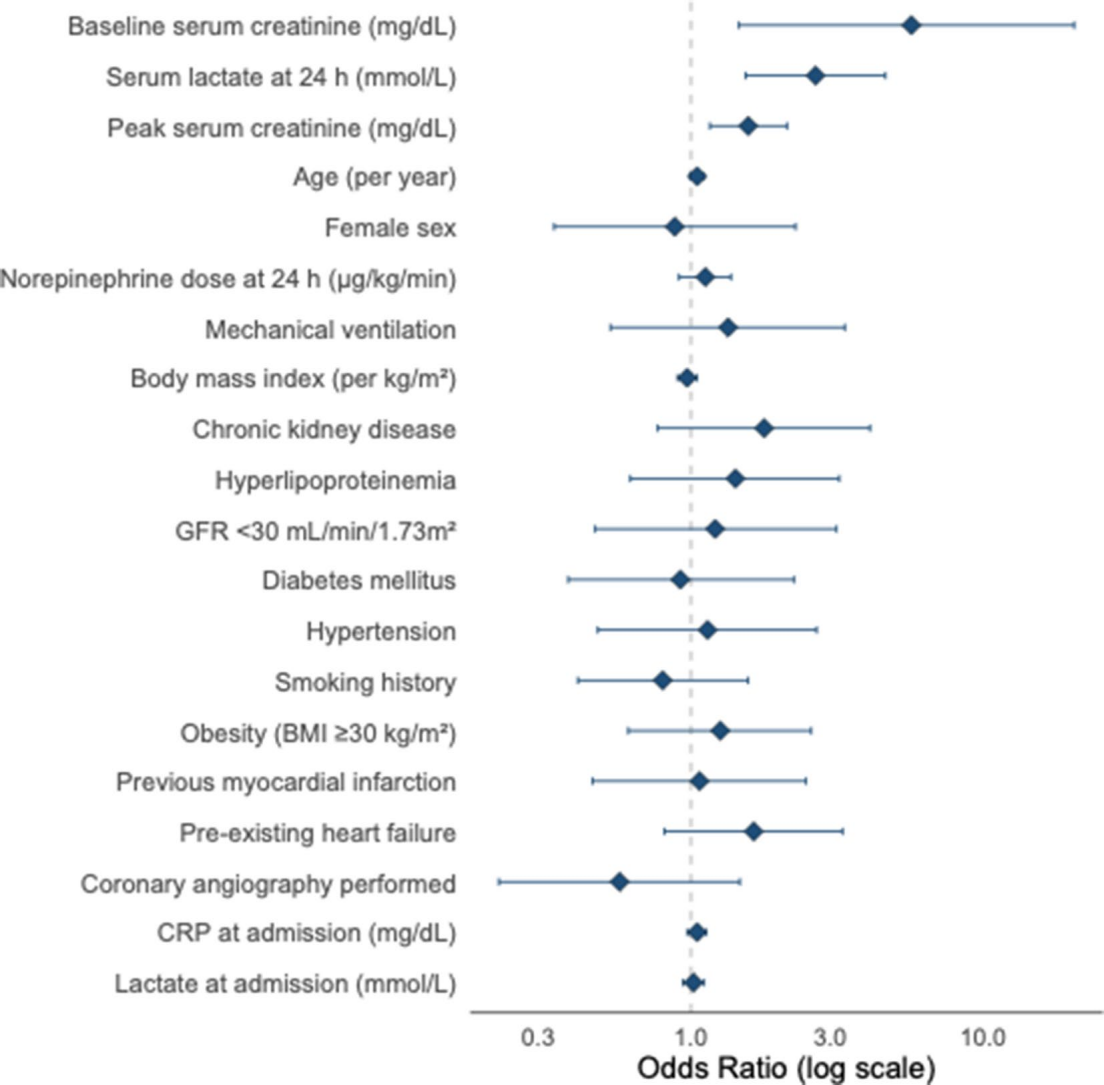
Independent predictors of early-onset acute kidney injury. Forest plot of adjusted odds ratios with 95% confidence intervals from a multivariable logistic regression model assessing factors associated with early-onset acute kidney injury (≤ 48 h) among patients with infarct-related cardiogenic shock. The vertical dashed line denotes an odds ratio of 1.0 (no association). Odds ratios are displayed on a logarithmic scale. Points indicate adjusted odds ratios; horizontal lines indicate 95% confidence intervals. Early-onset acute kidney injury was defined as AKI onset within 48 h after ICU admission. OR, odds ratio; CI, confidence interval; GFR, glomerular filtration rate; CRP, C-reactive protein

In multivariable logistic regression, two variables independently predicted the development of early-onset AKI. Higher baseline serum creatinine was associated with substantially increased risk (adjusted OR 5.68, 95% CI 1.46–20.48; *p* = 0.008), indicating renal vulnerability even before the onset of AKI. Elevated serum lactate at 24 h was an additional independent predictor (adjusted OR 2.67 per mmol/L, 95% CI 1.54–4.63; *p* < 0.001), reflecting persistent metabolic stress and hemodynamic compromise. Age showed a non-significant trend (*p* = 0.057), and no associations were observed for sex, BMI, norepinephrine requirements, mechanical ventilation, diabetes, hypertension, chronic kidney disease, or severely reduced baseline GFR (all *p* > 0.05). Full regression estimates appear in Table 3 and are visualized in Fig. 4.

**Table 3:** Independent predictors of early-onset AKI in CS

Variable	Odds Ratio (95% CI)	*P* value
Baseline serum creatinine (mg/dL)	5.68 (1.46–20.48)	**0.008**
Serum lactate at 24 h (mmol/L)	2.67 (1.54–4.63)	**< 0.001**
Peak serum creatinine (mg/dL)	1.57 (1.16–2.13)	**0.003**
Age (per year)	1.05 (0.99–1.11)	0.057
Female sex	0.88 (0.34–2.28)	0.79
Norepinephrine dose at 24 h (µg/kg/min)	1.12 (0.91–1.37)	0.28
Mechanical ventilation	1.34 (0.53–3.36)	0.52
Body mass index (per kg/m²)	0.97 (0.90–1.05)	0.45
Chronic kidney disease	1.78 (0.77–4.10)	0.18
Hyperlipoproteinemia	1.42 (0.62–3.22)	0.40
GFR < 30 mL/min/1.73 m²	1.21 (0.47–3.15)	0.69
Diabetes mellitus	0.92 (0.38–2.25)	0.86
Hypertension	1.14 (0.48–2.69)	0.77
Smoking history	0.80 (0.41–1.57)	0.52
Obesity (BMI ≥ 30 kg/m²)	1.26 (0.61–2.58)	0.53
Previous myocardial infarction	1.07 (0.46–2.47)	0.88
Pre-existing heart failure	1.64 (0.81–3.30)	0.17
Coronary angiography performed	0.57 (0.22–1.47)	0.24
CRP at admission (mg/dL)	1.05 (0.97–1.13)	0.21
Lactate at admission (mmol/L)	1.02 (0.94–1.11)	0.62

Multivariable logistic regression analysis identifying variables associated with AKI onset within 48 h. Only baseline creatinine and lactate at 24 h were independently associated with early AKI. *Abbreviations*: AKI, acute kidney injury; OR, odds ratio; CI, confidence interval; CKD, chronic kidney disease

### Survival analyses

**Fig. 5:**
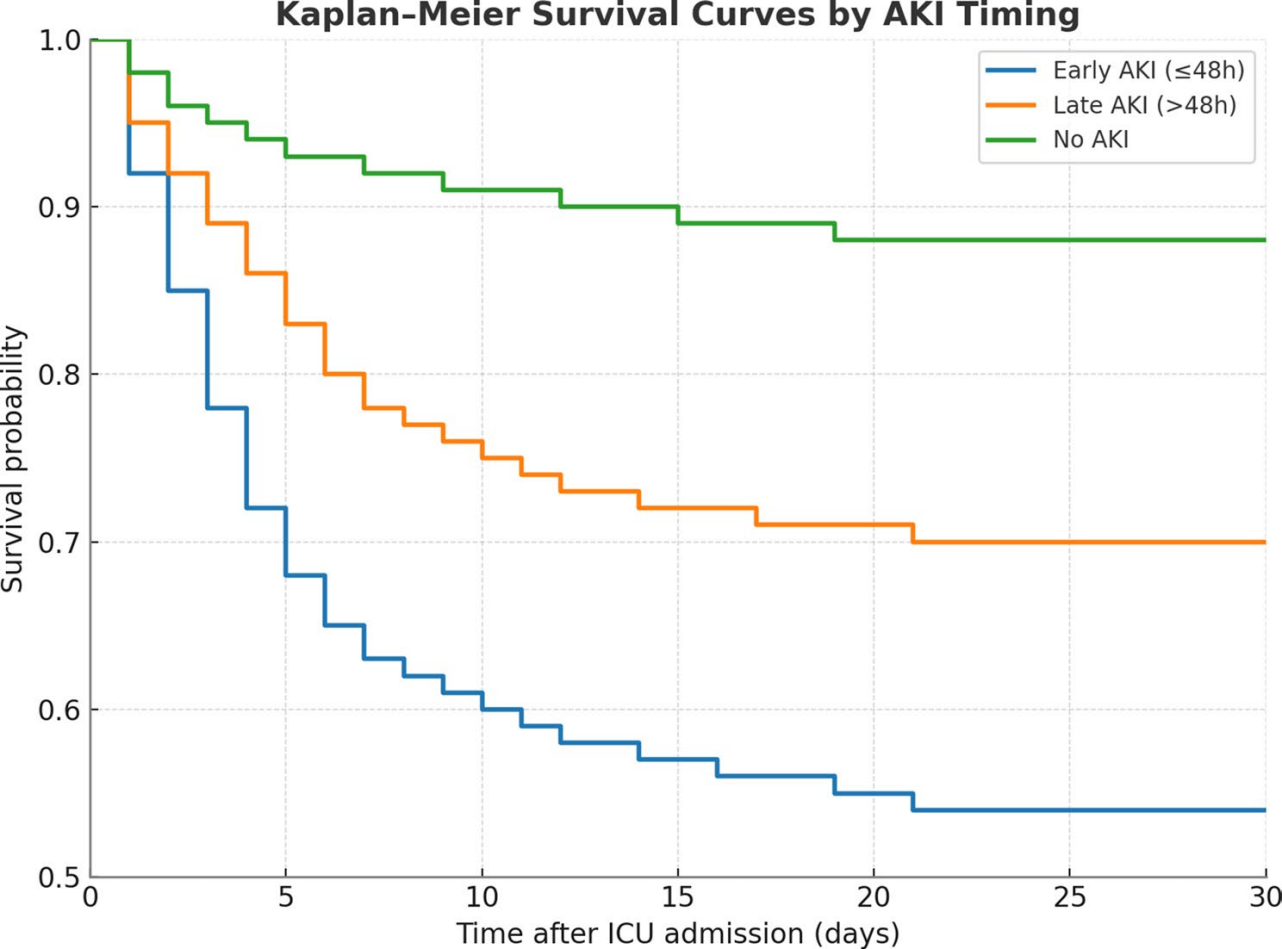
Kaplan–Meier survival analysis according to timing of AKI. Shown are Kaplan–Meier estimates of 30-day survival among patients with infarct-related cardiogenic shock, stratified by AKI status. Survival was lowest in patients with early AKI (≤ 48 h, blue), intermediate in those with late AKI (> 48 h, orange), and highest in patients without AKI (green) (log-rank *p* < 0.01)

Kaplan–Meier curves demonstrated markedly reduced survival in patients with early-onset AKI compared with late AKI and with no AKI (log-rank *p* < 0.01; Fig. 5). Early AKI remained independently associated with in-hospital mortality after adjustment for age, sex, baseline creatinine, 24-hour lactate and norepinephrine dose (adjusted OR 2.12, 95% CI 1.16–3.87; *p* = 0.015). The logistic model showed good calibration (Hosmer–Lemeshow *p* = 0.45) and moderate discrimination (AUC 0.72, 95% CI 0.66–0.78). In multivariable Cox regression, early AKI remained a strong predictor of mortality (HR 2.41, 95% CI 1.51–3.85; *p* < 0.001).

### Exploratory timing-based classification

**Fig. 6:**
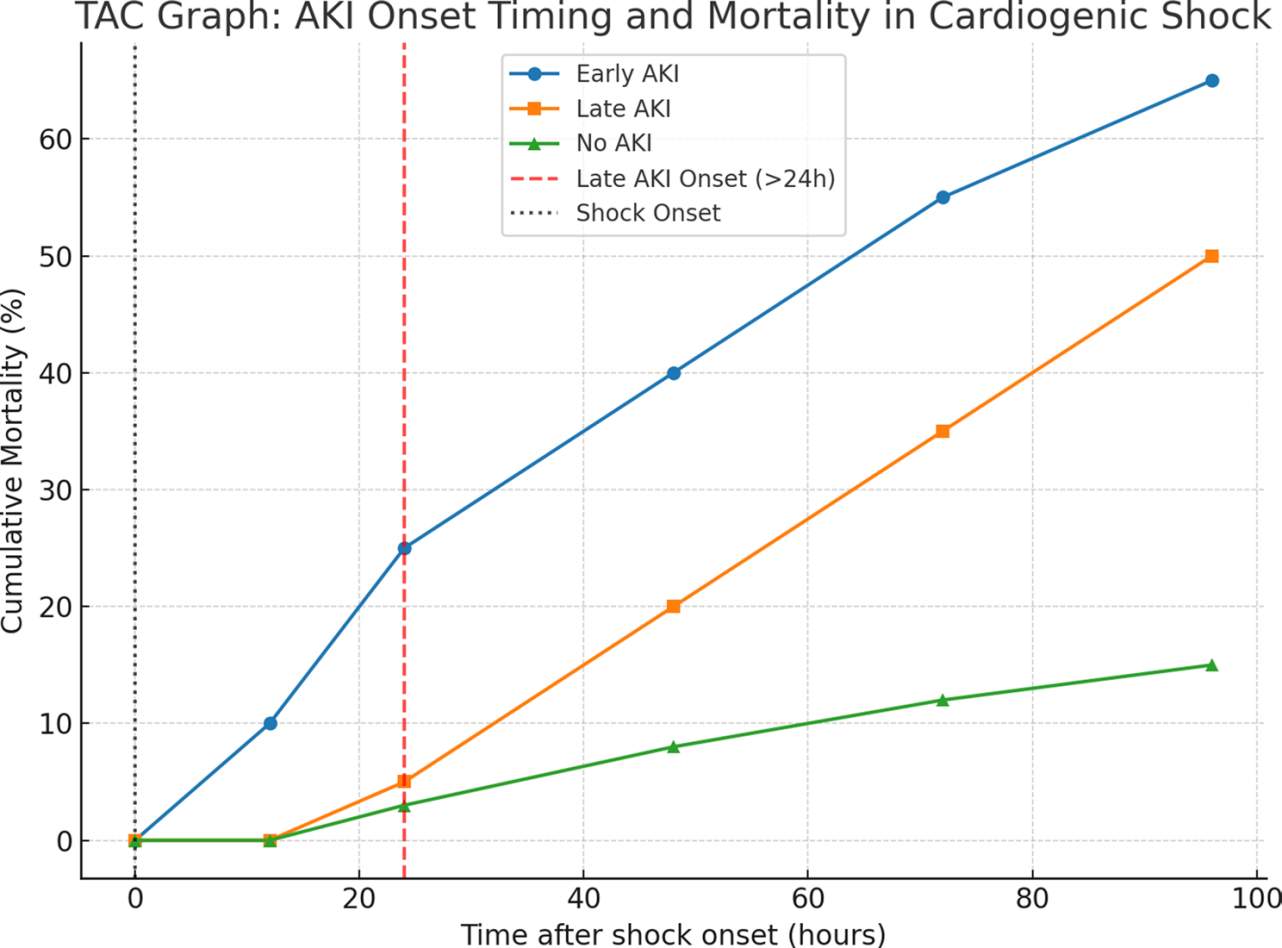
Cumulative mortality over time after cardiogenic shock onset, stratified by AKI timing. Early AKI (blue circles) denotes onset within 24 h of shock; Late AKI (orange squares) denotes onset after 24 h; No AKI (green triangles) represents patients without AKI. The vertical black dotted line indicates shock onset (0 h), and the vertical red dashed line marks the cut-off for late AKI onset (> 24 h)

Further stratification of AKI timing into early (< 24 h), intermediate (24–48 h), and late (> 48 h) onset revealed a stepwise decline in mortality (42.8%, 33.3%, and 18.7%, respectively), whereas patients without AKI had the lowest mortality (15.3%). In multivariable Cox regression, early AKI (< 24 h) remained strongly associated with in-hospital mortality (HR 2.87, 95% CI 1.73–4.76; *p* < 0.001), and intermediate AKI (24–48 h) showed a weaker but still significant association (HR 1.95, 95% CI 1.14–3.33; *p* = 0.015), whereas late AKI (> 48 h) was not significantly related to mortality. These temporal risk gradients are depicted in Fig. 6 and were preserved in propensity score–adjusted analyses (early vs. no AKI: HR 2.11, 95% CI 1.33–3.32; *p* < 0.001).

### Sensitivity and robustness analyses

To address the modest sample size, multivariable models were limited to a parsimonious set of prespecified covariates (age, sex, baseline creatinine, lactate, vasopressor dose). Effect estimates for the association between early AKI and in-hospital mortality remained consistent with the primary analysis (adjusted OR 2.45, 95% CI 1.21–4.96; *p* = 0.013). Firth’s penalized logistic regression produced comparable results (adjusted OR 2.39, 95% CI 1.14–4.85; *p* = 0.015), supporting robustness against small-sample bias. Nonparametric bootstrap resampling (1,000 iterations) yielded similar bias-corrected intervals (adjusted OR 2.41, 95% CI 1.17–4.89). The model including AKI timing showed good discrimination for mortality (AUC 0.77, 95% CI 0.70–0.84). Redefining late AKI as onset within 48–168 h did not alter findings, with no independent association with mortality (adjusted OR 1.12, 95% CI 0.54–2.35; *p* = 0.75).

## Discussion

This retrospective cohort of patients with infarct-related CS [42], the timing of AKI onset emerged as a clinically relevant prognostic factor [25]. Patients who developed AKI within the first 48 h—especially within < 24 h—had significantly higher in-hospital mortality compared to those with later AKI onset, despite similar baseline characteristics. Early AKI was also associated with increased lactate levels, higher vasopressor requirements, and more frequent use of RRT, pointing to a phenotype of more profound circulatory derangement in the early shock phase.

These findings reinforce the notion that early AKI reflects ischemic–hemodynamic insult, while late AKI may arise from multifactorial contributors such as inflammation, nephrotoxicity, and fluid overload [5, 9]. Prior studies in CS have consistently shown that early AKI portends worse outcomes and aligns closely with markers of systemic hypoperfusion [24, 25, 43]. Our data extend this knowledge by demonstrating that the AKI time course is not merely a chronological phenomenon, but a surrogate for distinct underlying pathophysiology. Beyond confirming previous observations, our study adds nuance by focusing on a homogeneous cohort of infarct-related CS and by differentiating very early AKI (≤ 24 h) from later occurrences. This temporal refinement highlights early AKI not only as a complication but also as a dynamic marker of circulatory collapse, thereby extending existing evidence.

From a mechanistic perspective, early AKI in CS likely represents acute ischemic tubular injury due to renal hypoperfusion, loss of autoregulation, and microvascular dysfunction [27, 44]. Our findings of higher serum lactate and norepinephrine dose at 24 h in the early AKI group support this hypothesis. Serum lactate is a robust biomarker of global tissue hypoperfusion and has been shown to correlate with both AKI incidence and mortality in critically ill patients [7, 13, 45]. Similarly, high vasopressor demand indicates persistent circulatory failure and may amplify renal ischemia through vasoconstrictive effects [1]. Our multivariable analysis identified baseline creatinine and serum lactate at 24 h as independent predictors of early AKI. The association with baseline creatinine suggests that patients with subclinical or overt chronic kidney dysfunction may have reduced renal reserve and are thus more susceptible to acute insults [24, 46]. This vulnerability may also reflect maladaptive repair responses and heightened sensitivity to systemic inflammation.

Interestingly, while norepinephrine dose at 24 h was higher in early AKI patients in univariate analysis, it did not independently predict early AKI in the adjusted model. This may reflect the complex interplay between vasopressor dose, timing, and underlying shock severity, as well as possible confounding by indication [9]. Likewise, mechanical ventilation, diabetes, hypertension, and sex did not independently associate with early AKI. Although sex differences in AKI susceptibility and outcomes have been reported [3, 47], their relevance may depend on contextual factors such as hormonal status, vascular reactivity, and timing of injury.

The clustering of AKI onset within the first 48 h supports the clinical distinction between early and late AKI phenotypes in CS. Early AKI appears to track with primary hemodynamic collapse and ischemia–reperfusion injury, whereas late AKI may reflect prolonged critical illness, secondary inflammation, or medication-induced nephrotoxicity [48, 49]. Recognizing these distinct trajectories could enable clinicians to tailor monitoring intensity, fluid resuscitation strategies, and timing of RRT initiation [5]. Beyond individual predictors, our data support efforts to redefine AKI phenotypes beyond static staging systems such as KDIGO, incorporating dynamic parameters such as timing, lactate clearance, and perfusion indices [43, 50]. For instance, lactate-guided resuscitation may help identify high-risk phenotypes and inform timely interventions in patients at risk of early AKI [30].

In infarct-related CS, the timing of AKI onset carries independent prognostic value. Early-onset AKI within 48 h identifies a hemodynamically compromised phenotype with excess mortality, underscoring the need to incorporate temporal patterns into future risk stratification and management strategies. The assessment of AKI in cardiogenic shock remains challenging. While urine output (UO) and creatinine clearance provide complementary information, both have practical limitations in critically ill patients [34, 48, 51]. In line with KDIGO recommendations, our analysis applied serum creatinine (SCr)–based criteria, as UO data were incomplete and potentially confounded by diuretics [52, 53]. In German ICUs, frequent blood sampling is routinely feasible under national health insurance coverage; in cases of oliguria, SCr was monitored several times per day, minimizing misclassification [38]. This approach ensured diagnostic consistency, though it may have underestimated AKI incidence and thus yielded conservative estimates of risk [54]. A planned subanalysis including patients with complete hourly UO records will further explore the prognostic impact of combined SCr–UO criteria. In infarct-related cardiogenic shock, the temporal pattern of AKI onset delineates distinct clinical phenotypes with prognostic relevance. Early-onset AKI within 48 h identifies a circulatory failure phenotype marked by hemodynamic instability, metabolic distress, and excess mortality, whereas later AKI more likely reflects the cumulative burden of critical illness. Recognizing these temporal trajectories may enable earlier identification of high-risk patients and more targeted renal-protective strategies.

The assessment of AKI in this context remains complex. While serum creatinine provides a consistent diagnostic anchor, the integration of urine output, dynamic renal biomarkers, and continuous perfusion monitoring could substantially improve phenotyping accuracy and timely detection. Building on these findings, ongoing analyses within our cohort aim to link AKI timing with recovery trajectories, urine output dynamics, renal replacement thresholds, and long-term outcomes. Ultimately, a time-sensitive, physiology-based framework of AKI in cardiogenic shock may enhance both prognostication and early intervention, paving the way for more personalized critical care.

### Limitations

This study has limitations. It was conducted at a single academic center, which may limit generalizability. The retrospective observational design precludes causal inference, and residual confounding cannot be excluded despite adjustment. Given the modest sample size, statistical power was limited, particularly for late-onset AKI, and the absence of an independent association with mortality should therefore be interpreted as hypothesis-generating rather than definitive. To mitigate overfitting, we applied parsimonious models and internal validation, but small-sample bias remains possible. Timing of AKI onset may have been misclassified because serum creatinine was measured daily rather than continuously. In addition, urine output–based KDIGO criteria could not be applied systematically across the entire cohort because complete 6-hour documentation was not consistently available in this retrospective setting. Important contributors to renal injury, including fluid balance, nephrotoxin exposure, were not systematically recorded. Sex-specific hormonal influences were not assessed, and long-term renal outcomes were beyond the scope of this analysis. Most patients had pre-existing CKD, reflecting the typical infarct-related CS population but reducing generalizability to those with preserved renal function. Using admission SCr as baseline may also have missed community-acquired or pre-hospital AKI. We deliberately avoided imputed baselines (e.g. MDRD with assumed eGFR = 75 mL/min/1.73 m²) or “lowest inpatient SCr” surrogates, which can overdiagnose AKI, particularly in CKD, and distort staging. This uniform approach ensured comparability but likely yielded conservative risk estimates. Another limitation is the exposure to iodinated contrast during angiography and PCI. Although this introduces potential contrast-associated AKI, the early temporal clustering of cases and the standardized use of low-osmolality contrast with periprocedural hydration make hemodynamic and ischemic injury the more plausible drivers of renal dysfunction.

## Conclusion

In infarct-related CS, the timing of AKI onset holds critical prognostic value. Early-onset AKI—particularly within 48 h—defines a high-risk phenotype marked by severe hemodynamic instability, metabolic stress, and substantially increased in-hospital mortality. Elevated lactate levels and reduced baseline renal function independently predicted early AKI, reflecting the interplay of global hypoperfusion and renal susceptibility. These findings support incorporating AKI timing into clinical risk models and underscore the need for early recognition and tailored renoprotective strategies to improve outcomes in this vulnerable population. While these findings reflect the real-world profile of infarct-related cardiogenic shock, the high prevalence of pre-existing chronic kidney disease in our cohort may limit generalizability to populations with preserved renal function. Prospective multicenter studies integrating urine output, dynamic biomarkers, and hemodynamic profiling are warranted to refine the temporal phenotyping of AKI in cardiogenic shock.

## Data Availability

The datasets generated and/or analyzed during the current study are available from the corresponding author upon reasonable request.
